# Revisiting Underapproximate Reachability for Multipushdown Systems

**DOI:** 10.1007/978-3-030-45190-5_21

**Published:** 2020-03-13

**Authors:** S. Akshay, Paul Gastin, S Krishna, Sparsa Roychowdhury

**Affiliations:** 8 Johannes Kepler University, Linz, Austria; 9 University of Birmingham, Birmingham, UK; 10 IIT Bombay, Mumbai, India; 11 ENS Paris-Saclay, Paris, France

**Keywords:** Multipushdown Systems, Underapproximate Reachability, Timed pushdown automata

## Abstract

Boolean programs with multiple recursive threads can be captured as pushdown automata with multiple stacks. This model is Turing complete, and hence, one is often interested in analyzing a restricted class which still captures useful behaviors. In this paper, we propose a new class of bounded underapproximations for multi-pushdown systems, which subsumes most existing classes. We develop an efficient algorithm for solving the under-approximate reachability problem, which is based on efficient fix-point computations. We implement it in our tool BHIM and illustrate its applicability by generating a set of relevant benchmarks and examining its performance. As an additional takeaway BHIM solves the binary reachability problem in pushdown automata. To show the versatility of our approach, we then extend our algorithm to the timed setting and provide the first implementation that can handle timed multi-pushdown automata with closed guards.

## 1 Introduction

The reachability problem for pushdown systems with multiple stacks is known to be undecidable. However, multi-stack pushdown automata (MPDA hereafter) represent a theoretically concise and analytically useful model of multi-threaded recursive programs with shared memory. As a result, several previous works in the literature have proposed different under-approximate classes of behaviors of MPDA that can be analyzed effectively, such as *Round Bounded*, *Scope Bounded*, *Context Bounded* and *Phase Bounded* [18,19,27,14,20,28]. From a practical point of view, these underapproximations have led to efficient tools including, GetaFix [21], SPADE [23]. It has also been argued (e.g., see [24]) that such bounded underapproximations suffice to find several bugs in practice. In many such tools efficient fix-point techniques are used to speed-up computations.

We extend known fix-point based approaches by developing a new algorithm that can handle a larger class of bounded underapproximations than the well-known bounded context and bounded scope underapproximations for

⋆ Partly supported by UMI ReLaX, DST/CEFIPRA/INRIA project EQuaVe & TCS.

multi-pushdown systems while remaining efficiently implementable. Our algorithm works for a new class of underapproximate behaviors called *hole bounded* behaviors, which subsumes context/scope bounded underapproximations, and is orthogonal to phase bounded underapproximations. A "hole" is a maximal sequence of push operations of a fixed stack, interspersed with well-nested sequences of any stack. Thus, in a sequence $\alpha = \beta\gamma$ where $\beta = [push_1(push_2 push_3\, pop_3 pop_2) push_1(push_3 pop_3)]^{10}$ and $\gamma = push_2 push_1 pop_2 pop_1 (pop_1)^{20}$, $\beta$ is a hole with respect to stack 1. The suffix $\gamma$ has 2 holes (the $push_2$ and the $push_1$). Thus we say that $\alpha$ is 3-hole bounded. On the other hand, the number of context switches (and scope bound) in $\alpha$ is $> 50$. A ($k$-)hole bounded sequence is one such, where, at any point of the computation, the number of "open" holes are bounded at this point (by $k$). We show that the class of hole bounded sequences subsumes most of the previously defined classes of underapproximations and is, in fact, contained in the very generic class of tree-width bounded sequences. This immediately shows decidability of the reachability problem for our class.

Analyzing the more generic class of tree-width bounded sequences is often much more difficult; for instance, building bottom-up tree automata for this purpose does not scale very well as it explores a large (and often useless) state space. Our technique is radically different from using tree automata. Under the hole bounded assumption, we pre-compute information regarding well-nested sequences and holes using fix-point computations and use them in our algorithm. Using efficient data structures to implement this approach, we develop a tool (BHIM) for Bounded Hole reachability in Multi-stack pushdown systems.

**Highlights of BHIM.**

• Two significant aspects of the fix-point approach in BHIM are: (i) we efficiently solve the binary reachability problem for pushdown automata. i.e., BHIM computes all pairs of states $(s, t)$ such that $t$ is reachable from $s$ with empty stacks. This allows us to go beyond reachability and handle some liveness questions; (ii) we pre-compute the set of pairs of states that are endpoints of holes. This allows us to greatly limit the search for an accepting run.

• While the fix-point approach solves (binary) reachability efficiently, it does not a priori produce a witness of reachability. We remedy this situation by proposing a backtracking algorithm, which cleverly uses the computations done in the fix-point algorithm, to generate a witness efficiently.

• BHIM is parametrized with respect to the hole bound: if non-emptiness can be checked or witnessed by a well-nested sequence (this is an easy witness and BHIM looks for easy witnesses first, then gradually increases complexity, if no easy witness is found), then it is sufficient to have the hole bound 0. Increasing this complexity measure as required to certify non-emptiness gives an efficient implementation, in the sense that we search for harder witnesses only when no easier witnesses (w.r.t this complexity measure) exist. In examples described in the experimental section, a small (less than 4) bound suffices and we expect this to be the case for most practical examples.

• Finally, we extend our approach to handle timed multi-stack pushdown systems. This shows the versatility of our approach and also requires us to solve several technical challenges which are specific to the timed setting. Implementing this approach in BHIM makes it, to the best of our knowledge, the first tool that can analyze timed multi-stack pushdown automata with closed guards.

We analyze the performance of BHIM in practice, by considering benchmarks from the literature, and generating timed variants of some of them. One of our benchmarks is a variant of the Bluetooth example [11,23], where BHIM was able to catch a known race detection error. Another interesting benchmark is a model of a parameterized multiple producer consumer example, having parameters $M, N$ on the quantities of two items $A, B$ produced. Here, BHIM could detect bugs by finding witnesses having just 2 holes, while, it is unlikely that existing tools working on scope/context bounded underapproximations can handle them as the number of scope/context switches is dependent on $M, N$ (in fact, it is twice the least common multiple of $M$ and $N$). In the timed setting, one of the main challenges has been the unavailability of timed benchmarks; even in the untimed setting, many benchmarks were unavailable due to their proprietary nature. Due to lack of space, proofs, technical details and parametric plots of experiments are in [4].

**Related Work**. Among other under-approximations, scope bounded [27] subsumes context and round bounded underapproximations, and it also paves path for GetaFix [21], a tool to analyze recursive (and multi-threaded) boolean programs. As mentioned earlier hole boundedness strictly subsumes scope boundedness. On the other hand, GetaFix uses symbolic approaches via BDDs, which is orthogonal to the improvements made in this paper. Indeed, our next step would be to build a symbolic version of BHIM which extends the hole-bounded approach to work with symbolic methods. Given that BHIM can already handle synthetic examples with 12-13 holes (see [4]), we expect this to lead to even more drastic improvements and applicability. For sequential programs, a summary-based algorithm is used in [21]; summaries are like our well-nested sequences, except that well-nested sequences admit contexts from different stacks unlike summaries. As a result, our class of bounded hole behaviors generalizes summaries. Many other different theoretical results like phase bounded [18], order bounded [8] which gives interesting underapproximations of MPDA, are subsumed in tree-width bounded behaviors, but they do not seem to have practical implementations. Adding real-time information to pushdown automata by using clocks or timed stacks has been considered, both in the discrete and dense-timed settings. Recently, there has been a flurry of theoretical results in the topic [10,1,2,5,6]. However, to the best of our knowledge none of these algorithms have been successfully implemented (except [6] which implements a tree-automata based technique for single-stack timed systems) for multi-stack systems. One reason is that these algorithms do not employ scalable fix-point based techniques, but instead depend on region automaton-based search or tree automata-based search techniques.

## 2 Underapproximations in MPDA

A multi-stack pushdown automaton (MPDA) is a tuple $M = (\mathcal{S}, \Delta, s_0, \mathcal{S}_f, n, \Sigma, \Gamma)$ where, $\mathcal{S}$ is a finite non-empty set of locations, $\Delta$ is a finite set of transitions, $s_0 \in \mathcal{S}$ is the initial location, $\mathcal{S}_f \subseteq \mathcal{S}$ is a set of final locations, $n \in \mathbb{N}$ is the number of stacks, $\Sigma$ is a finite input alphabet, and $\Gamma$ is a finite stack alphabet which contains $\perp$. A transition $t \in \Delta$ can be represented as a tuple $(s, \mathsf{op}, a, s')$, where, $s, s' \in \mathcal{S}$ are respectively, the source and destination locations of the transition $t$, $a \in \Sigma$ is the label of the transition, and $\mathsf{op}$ is one of the following operations (1) $\mathsf{nop}$, or no stack operation, (2) $(\downarrow_i \alpha)$ which pushes $\alpha \in \Gamma$ onto stack $i \in \{1, 2, \ldots, n\}$, (3) $(\uparrow_i \alpha)$ which pops stack $i$ if the top of stack $i$ is $\alpha \in \Gamma$.

For a transition $t = (s, \mathsf{op}, a, s')$ we write $\mathsf{src}(t) = s, \mathsf{tgt}(t) = s'$ and $\mathsf{op}(t) = \mathsf{op}$. At the moment we ignore the action label $a$ but this will be useful later when we go beyond reachability to model checking. A *configuration* of the MPDA is a tuple $(s, \lambda_1, \lambda_2, \ldots, \lambda_n)$ such that, $s \in \mathcal{S}$ is the current location and $\lambda_i \in \Gamma^*$ represents the current content of $i^{th}$ stack. The semantics of the MPDA is defined as follows: a run is accepting if it starts from the initial state and reaches a final state with all stacks empty. The language accepted by a MPDA is defined as the set of words generated by the accepting runs of the MPDA. Since the reachability problem for MPDA is Turing complete, we consider under-approximate reachability.

A sequence of transitions is called **complete** if each push in that sequence has a matching pop and vice versa. A **well-nested** sequence denoted $ws$ is defined inductively as follows: a possibly empty sequence of $\mathsf{nop}$-transitions is $ws$, and so is the sequence $t\ ws\ t'$ where $\mathsf{op}(t) = (\downarrow_i \alpha)$ and $\mathsf{op}(t') = (\uparrow_i \alpha)$ are a matching pair of push and pop operations of stack $i, \forall i \in \{1 \ldots n\}$. Finally the concatenation of two well-nested sequences is a well-nested sequence, i.e., they are closed under concatenation. The set of all well-nested sequences defined by an MPDA is denoted WS. If we visualize this by drawing edges between pushes and their corresponding pops, well-nested sequences have no crossing edges, as in  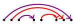 and 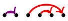 , where we have two stacks, depicted with red and violet edges. We emphasize that a well-nested sequence can have well-nested edges from any stack. In a sequence $\sigma$, a push (pop) is called a **pending** push (pop) if its matching pop (push) is not in the same sequence $\sigma$.

**Bounded Underapproximations**. As mentioned in the introduction, different bounded under-approximations have been considered in the literature to get around the Turing completeness of MPDA. During a computation, a context is a sequence of transitions where only one stack or no stack is used. In *context bounded* computations the number of contexts are bounded [25]. A *round* is a sequence of (possibly empty) contexts for stacks $1, 2, \ldots, n$. *Round bounded* computations restrict the total number of rounds allowed [19,5,6]. *Scope bounded* computations generalize bounded context computations. Here, the context changes within any push and its corresponding pop is bounded [19,20,28]. A *phase* is a contiguous sequence of transitions in a computation, where we restrict pop to only one stack, but there are no restrictions on the pushes [18]. A phase bounded computation is one where the number of phase changes is bounded.

**Tree-width**. A generic way of looking at them is to consider classes which have a bound on the tree-width [22]. In fact, the notions of split-width/cliquewidth/tree-width of communicating finite state machines/timed push down systems has been explored in [3], [13]. The behaviors of the underlying system are then represented as graphs. It has been shown in these references that if the family of graphs arising from the behaviours of the underlying system (say $S$) have a bounded tree-width, then the reachability problem is decidable for $S$ via, tree-automata. However, this does not immediately give rise to an efficient implementation. The tree-automata approach usually gives non-deterministic or bottom-up tree automata, which when implemented in practice (see [6]) tend to blow up in size and explore a large and useless space. Hence there is a need for efficient algorithms, which exist for more specific underapproximations such as context-bounded (leading to fix-point algorithms and their implementations [21]).

## 2.1 A new class of under-approximations

Our goal is to bridge the gap between having practically efficient algorithms and handling more expressive classes of under-approximations for reachability of multi-stack pushdown systems. To do so, we define a bounded approximation which is expressive enough to cover previously defined practically interesting classes (such as context bounded etc), while at the same time allowing efficient decidable reachability tests, as we will see in the next section.

**Definition 1.** *(Holes). Let $\sigma$ be complete sequence of transitions, of length $n$ in a* MPDA*, and let $ws$ be a well-nested sequence.*

- *A* ***hole*** *of stack $i$ is a maximal factor of $\sigma$ of the form $(\downarrow_i ws)^+$, where $ws \in \mathsf{WS}$. The maximality of the hole of stack $i$ follows from the fact that any possible extension ceases to be a hole of stack $i$; that is, the only possible events following a maximal hole of stack $i$ are a push $\downarrow_j$ of some stack $j \neq i$, or a pop of some stack $j \neq i$. In general, whenever we speak about a hole, the underlying stack is clear.*
- *A push $\downarrow_i$ in a hole (of stack $i$) is called a pending push at (i.e., just before) a position $x \leq n$, if its matching pop occurs in $\sigma$ at a position $z > x$.*
- *A hole (of stack $i$) is said to be* ***open*** *at a position $x \leq n$, if there is a pending push $\downarrow_i$ of the hole at $x$. Let $\#_x(\mathsf{hole})$ denote the number of open holes at position $x$. The* ***hole bound*** *of $\sigma$ is defined as $\max_{1 \leq x \leq |\sigma|} \#_x(\mathsf{hole})$.*
- *A* hole segment *of stack $i$ is a prefix of a hole of stack $i$, ending in a $ws$, while an* atomic hole segment *of stack $i$ is just the segment of the form $\downarrow_i ws$.*

As an example, consider the sequence $\sigma$ in Figure 1 of transitions of a MPDA having stacks 1,2 (denoted respectively red and blue). We use superscripts for

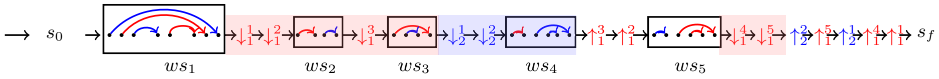

**Figure 1.** A run $\sigma$ with 2 holes (2 red patches) of the red stack and 1 hole (one blue patch) of the blue stack.

each push, pop of each stack to distinguish the $i$th push, $j$th pop and so on of each stack. There are two holes of stack 1 (red stack) denoted by the red patches, and one hole of stack 2 (blue stack) denoted by the blue patch. The subsequence $\downarrow_1^1\downarrow_1^2$ $ws_2$ of the first hole is not a maximal factor, since it can be extended by $\downarrow_1^3$ $ws_3$ in the run $\sigma$, extending the hole. Consider the position in $\sigma$ marked with $\downarrow_2^1$. At this position, there is an open hole of the red stack (the first red patch), and there is an open hole of the blue stack (the blue patch). Likewise, at the position $\uparrow_1^5$, there are 2 open holes of the red stack (2 red patches) and one open hole of the blue stack 2 (the blue patch). The hole bound of $\sigma$ is 3. The green patch consisting of $\uparrow_1^3$, $\uparrow_1^2$ and $ws_5$ is a pop-hole of stack 1. Likewise, the pops $\uparrow_2^2$, $\uparrow_1^5$, $\uparrow_2^1$ are all pop-holes (of length 1) of stacks 2,1,2 respectively.

**Definition 2.** (Hole Bounded Reachability Problem) *Given a* MPDA *and $K \in \mathbb{N}$, the $K$-hole bounded reachability problem is the following: Does there exist a $K$-hole bounded accepting run of the* MPDA*?*

**Proposition 1.** *The tree-width of $K$-hole bounded* MPDA *behaviors is at most $(2K+3)$.*

With this, from [22][5][6], decidability and complexity follow. Thus,

**Corollary 1.** *The $K$-hole bounded reachability problem for* MPDA *is decidable in $\mathcal{O}(|\mathcal{M}|^{2K+3})$ where, $\mathcal{M}$ is the size of the underlying* MPDA*.*

Next, we turn to the expressiveness of this class with respect to the classical underapproximations of MPDA: first, the **hole** bounded class strictly subsumes **scope** bounded which already subsumes **context** bounded and **round** bounded classes. Also **hole** bounded MPDA and **phase** bounded MPDA are orthogonal.

**Proposition 2.** *Consider a* MPDA *$M$. For any $K$, let $L_K$ denote a set of sequences accepted by $M$ which have number of rounds or number of contexts or scope bounded by $K$. Then there exists $K' \leq K$ such that $L_K$ is $K'$ hole bounded. Moreover, there exist languages which are $K$ hole bounded for some constant $K$, which are not $K'$ round or context or scope bounded for any $K'$. Finally, there exists a language which is accepted by phase bounded* MPDA *but not accepted by hole bounded* MPDA *and vice versa.*

*Proof.* We first recall that if a language $L$ is $K$-round, or $K$-context bounded, then it is also $K'$-scope bounded for some $K' \leq K$ [20,19]. Hence, we only show that scope bounded systems are subsumed by hole bounded systems.

Let $L$ be a $K$-scope bounded language, and let $M$ be a MPDA accepting $L$. Consider a run $\rho$ of $w \in L$ in $M$. Assume that at any point $i$ in the run $\rho$, $\#_i(\texttt{holes}) = k'$, and towards a contradiction, let, $k' > K$. Consider the leftmost open hole in $\rho$ which has a pending push $\downarrow_p$ whose pop $\uparrow_p$ is to the right of $i$. Since $k' > K$ is the number of open holes at $i$, there are at least $k' > K$ context changes in between $\downarrow_p$ and $\uparrow_p$. This contradicts the $K$-scope bounded assumption, and hence $k' \leq K$.

To show the strict containment, consider the visibly pushdown language [7] given by $L^{bh} = \{a^n b^n (a^{p_1} c^{p_1+1} b^{p'_1} d^{p'_1+1} \cdots a^{p_n} c^{p_n+1} b^{p'_n} d^{p'_n+1}) \mid n, p_1, p'_1, \ldots, p_n, p'_n \in$

$\mathbb{N}\}$. A possible word $w \in L^{bh}$ is $a^3b^3\ a^2c^3b^2d^3\ a^2c^3bd^2\ ac^2bd^2$ with $a, b$ representing push in stack 1,2 respectively and $c, d$ representing the corresponding matching pop from stack 1,2. A run $\rho$ accepting the word $w \in L^{bh}$ will start with a sequence of pushes of stack 1 followed by another sequence of pushes of stack 2. Note that, the number of the pushes $n$ is same in both stacks. Then there is a group $G$ consisting of a well-nested sequence of stack 1 (equal $a$ and $c$) followed by a pop of the stack 1 (an extra $c$), another well-nested sequence of stack 2 (equal $b$ and $d$) and a pop of the stack 2 (an extra $d$), repeated $n$ times. From the definition of the `hole`, the total number of holes required in $G$ is 0. But, we need 1 hole for the sequence of $a$'s and another for the sequence of $b$'s at the beginning of the run, which creates at most 2 holes during the run. Thus, the hole bound for any accepting run $\rho$ is 2, and the language $L^{bh}$ is 2-hole bounded.

However, $L^{bh}$ is not $k$-scope bounded for any $k$. Indeed, for each $m \geq 1$, consider the word $w_m = a^m b^m (ac^2bd^2)^m \in L^{bh}$. It is easy to see that $w_m$ is $2m$-scope bounded (the matching $c, d$ of each $a, b$ happens $2m$ context switches later) but not $k$-scope bounded for $k < 2m$. It can be seen that $L^{bh}$ is not $k$-phase bounded either. Finally, $L' = \{(ab)^n c^n d^n \mid n \in \mathbb{N}\}$ with $a, b$ and $c, d$ respectively being push and pop of stack 1,2 is not hole-bounded but 2-phase bounded. □

## 3 A Fix-point Algorithm for Hole Bounded Reachability

In the previous section, we showed that hole-bounded underapproximations are a decidable subclass for reachability, by showing that this class has a bounded tree-width. However, as explained in the introduction, this does not immediately give a fix-point based algorithm, which has been shown to be much more efficient for other more restricted sub-classes, e.g., context-bounded. In this section, we provide such a fix-point based algorithm for the hole-bounded class and explain its advantages. Later we discuss its versatility by showing extensions and evaluating its performance on a suite of benchmarks.

We describe the algorithm in two steps: first we give a simple fix-point based algorithm for the problem of 0-hole or *well-nested reachability*, i.e, reachability by a well-nested sequence without any holes. For the 0-hole case, our algorithm computes the *reachability relation*, also called the *binary reachability problem [15]*. That is, we accept all pairs of states $(s, s')$ such that there is a well-nested run from $s$ with empty stack to $s'$ with empty stack. Subsequently, we combine this binary reachability for well-nested sequences with an efficient graph search to obtain an algorithm for $K$-hole bounded reachability.

**Binary well-nested reachability for** MPDA. Note that single stack PDA are a special case, since all runs are indeed well-nested.

1. **Transitive Closure**: Let $\mathcal{R}$ be the set of tuples of the form $(s_i, s_j)$ representing that state $s_j$ is reachable from state $s_i$ via a nop discrete transition. Such a sequence from $s_i$ to $s_j$ is trivially *well-nested.* We take the TransitiveClosure of $\mathcal{R}$ using Floyd-Warshall algorithm [12]. The resulting set $\mathcal{R}_c$ of tuples answers the binary reachability for finite state automata (no stacks).

**Algorithm 1:** Algorithm for Emptiness Checking of hole bounded MPDA

```
Function IsEmpty(M = (S, Δ, s_0, S_f, n, Σ, Γ), K):
    Result: True or False
    WR := WellNestedReach(M); \\Solves binary reachability for pushdown system
    if some (s_0, s_1) ∈ WR with s_1 ∈ S_f then
        return False;
    forall i ∈ [n] do
        AHS_i := ∅; Set_i := ∅;
        forall (s, ↓_i(α), a, s_1) ∈ Δ and (s_1, s') ∈ WR do
            AHS_i := AHS_i ∪ {(i, s, α, s')}; Set_i := Set_i ∪ {(s, s')};
        HS_i := {(i, s, s') | (s, s') ∈ TransitiveClosure(Set_i)};
    μ := [s_0]; μ.NumberOfHoles := 0;
    SetOfLists_new := {μ}; SetOfLists := ∅;
    do
        SetOfLists := SetOfLists ∪ SetOfLists_new;
        SetOfLists_todo := SetOfLists_new; SetOfLists_new := ∅;
        forall μ' ∈ SetOfLists_todo do
            if μ'.NumberOfHoles < K then
                forall i ∈ [n] do
                    \\ Add hole for stack i
                    SetOfLists_h := AddHole_i(μ', HS_i) \ SetOfLists;
                    SetOfLists_new := SetOfLists_new ∪ SetOfLists_h;
            if μ'.NumberOfHoles > 0 then
                forall i ∈ [n] do
                    \\ Add pop for stack i
                    SetOfLists_p := AddPop_i(μ', M, AHS_i, HS_i, WR) \ SetOfLists;
                    SetOfLists_new := SetOfLists_new ∪ SetOfLists_p;
                    forall μ_3 ∈ SetOfLists_p do
                        if μ_3.last ∈ S_f and μ_3.NumberOfHoles = 0 then
                            return False; \\If reached destination state
    while SetOfLists_new ≠ ∅;
    return True;
```

2. **Push-Pop Closure**: For stack operations, consider a push transition on some stack (say stack $i$) of symbol $\gamma$, enabled from a state $s_1$, reaching state $s_2$. If there is a matching pop transition from a state $s_3$ to $s_4$, which pops the same stack symbol $\gamma$ from the stack $i$ and if we have $(s_2, s_3) \in \mathcal{R}_c$, then we can add the tuple $(s_1, s_4)$ to $\mathcal{R}_c$. The function WellNestedReach repeats this process and the transitive closure described above until a fix-point is reached. Let us denote the resulting set of tuples by WR. Thus,

**Lemma 1.** *$(s_1, s_2) \in$ WR iff $\exists$ a well-nested run in the* MPDA *from $s_1$ to $s_2$.*

**Beyond well-nested reachability**. A naive algorithm for $K$-hole bounded reachability for $K > 0$ is to start from the initial state $s_0$, and do a Breadth First Search (BFS), nondeterministically choosing between extending with a well-nested segment, creating hole segments (with a pending push) and closing hole segments (using pops). We accept when there are no open hole segments and reach a final state; this gives an exponential time algorithm. Given the exponential dependence on the hole-bound $K$ (Corollary 1), this exponential blowup is unavoidable in the worst case, but we can do much better in practice. In particular, the naive algorithm makes arbitrary non-deterministic choices resulting in a blind exploration of the BFS tree.

In this section, we use the binary well-nested reachability algorithm as an efficient subroutine to limit the search in BFS to its reachable part (note that this is quite different from DFS as well since we do not just go down one path). The crux is that at any point, we create a new hole for stack $i$, *only* when (i) we know that we cannot reach the final state without creating this hole and (ii) we know that we can close all such holes which have been created. Checking (i) is easy, since we just use the WR relation for this. Checking (ii) blindly would correspond to doing a DFS; however, we precompute this information and simply look it up, resulting in a constant time operation after the precomputation.

**Precomputing hole information.** Recall that a *hole* of stack $i$ is a maximal sequence of the form $(\downarrow_i ws)^+$, where $ws$ is a well-nested sequence and $\downarrow_i$ represents a push of stack $i$. A *hole segment* of stack $i$ is a prefix of a hole of stack $i$, ending in a $ws$, while an *atomic hole segment* of stack $i$ is just the segment of the form $\downarrow_i ws$. A *hole-segment* of stack $i$ which starts from state $s$ in the MPDA and ends in state $s'$, can be represented by the triple $(i, s, s')$, that we call a *hole triple*. We compute the set $HS_i$ of all hole triples $(i, s, s')$ such that starting at $s$, there is a hole segment of stack $i$ which ends at state $s'$, as detailed in lines (5-9) of Algorithm 1. In doing so, we also compute the set $AHS_i$ of all atomic hole segments of stack $i$ and store them as tuples of the form $(i, s_p, \alpha, s_q)$ such that $s_p$ and $s_q$ are the MPDA states respectively at the left and right end points of an atomic hole segment of stack $i$, and $\alpha$ is the symbol pushed on stack $i$ ($s_p \xrightarrow{\downarrow_i(\alpha)ws} s_q$).

**A guided BFS exploration.** We start with a list $\mu_0 = [s_0]$ consisting of the initial state and construct a BFS exploration tree whose nodes are lists of bounded length. A list is a sequence of states and hole triples representing a $K$-hole bounded run in a concise form. If $H_i$ represents a hole triple for stack $i$, then a list is a sequence of the form $[s, H_i, H_j, H_k, H_i, \ldots, H_\ell, s']$. The simplest kind of list is a single state $s$. For example, a list with 3 holes of stacks $i, j, k$ is $\mu = [s_0, (i, s, s'), (j, r, r'), (k, t, t'), t'']$. The hole triples (in red) denote open holes in the list. The maximum number of open holes in a list is bounded, making the length of the list also bounded. Let $\mathsf{last}(\mu)$ represent the last element of the list $\mu$. This is always a state. For a node $v$ storing list $\mu$ in the BFS tree, if $v_1, \ldots v_k$ are its children, then the corresponding lists $\mu_1, \ldots \mu_k$ are obtained by extending the list $\mu$ by one of the following operations:

1. **Extend $\mu$ with a hole**. Assume there is a hole of some stack $i$, which starts at $\mathsf{last}(\mu) = s$, and ends at $s'$. If the list at the parent node $v$ is $\mu = [\ldots, s]$, then for all $(i, s, s') \in HS_i$, we obtain the list $\mathsf{trunc}(\mu) \cdot \mathsf{append}[(i, s, s'), s']$ at the child node (i.e., we remove the last element $s$ of $\mu$, then append to this list the hole triple $(i, s, s')$, followed by $s'$).
2. **Extend $\mu$ with a pop**. Suppose there is a transition $t = (s_k, \uparrow_i(\alpha), a, s'_k)$ from $\mathsf{last}(\mu) = s_k$, where $\mu$ is of the form $[s_0, \ldots, (h, u, v), (i, s, s'), (j, t, t') \ldots, s_k]$, such that there is no hole triple of stack $i$ after $(i, s, s')$, we extend the run by matching this pop (with its push). However, to obtain the last pending push of stack $i$ corresponding to this hole, just $HS_i$ information is not enough since we also need to match the stack content. Instead, we check if we can split the hole $(i, s, s')$ into (1) a hole triple $(i, s, s_a) \in HS_i$, and (2) a tuple $(i, s_a, \alpha, s') \in AHS_i$. If both (1) and (2) are possible, then the pop transition $t$ corresponds to the last pending push of the hole $(i, s, s')$. $t$ indeed matches the pending push recorded in the atomic hole $(i, s_a, \alpha, s')$ in $\mu$, enabling the firing of transition $t$ from the state $s_k$, reaching $s'_k$. In this case, we add the child node with the list $\mu'$ obtained from $\mu$ as follows. We replace (i) $s_k$ with $s'_k$, and (ii) $(i, s, s')$ with $(i, s, s_a)$, respectively signifying firing of the transition $t$ and the "shrinking" of the hole, by shifting the end point of the hole segment to the left. When we obtain the hole triple $(i, s, s)$ (the start and end points of the hole segment coincide), we may have uncovered the last pending push and thereby "closed" the hole segment completely. At this point, we may choose to remove $(i, s, s)$ from the list, obtaining $[s_0, \dots, (h, u, v), (j, t, t') \dots, s'_k]$. For every such $\mu' = [s_0, \dots, (h, u, v), (i, s, s_a), (j, t, t'), \dots, s'_k]$ and all $(s'_k, s_m) \in WS$ we also extend $\mu'$ to $\mu'' = [s_0, \dots, (h, u, v), (i, s, s_a), (j, t, t'), \dots, s_m]$. Notice that the size of the list in the child node obtained on a pop, is either the same as the list in the parent, or is smaller.

The number of lists is bounded since the number of states and the length of the lists are bounded. The BFS exploration tree will thus terminate. Combining the above steps gives us Algorithm 1, whose correctness gives us:

**Theorem 1.** *Given a* MPDA *and a positive integer $K$, Algorithm 1 terminates and answers "false" iff there exists a $K$-hole bounded accepting run of the* MPDA.

**Complexity of the Algorithm**. The maximum number of states of the system is $|\mathcal{S}|$. The time complexity of transitive closure is $\mathcal{O}(|\mathcal{S}|^3)$, using a Floyd-Warshall implementation. The time complexity of computing WellNestedReach which uses the transitive closure, is $\mathcal{O}(|\mathcal{S}|^5) + \mathcal{O}(|\mathcal{S}|^2 \times (|\Delta| \times |\mathcal{S}|))$. To compute $AHS$ for $n$ stacks the time complexity is $\mathcal{O}(n \times |\Delta| \times |\mathcal{S}|^2)$ and to compute $HS$ for $n$ stacks the complexity is $\mathcal{O}(n \times |\mathcal{S}|^2)$. For multistack systems, each list keeps track of (i) the number of hole segments($\leq K$), and (ii) information pertaining to holes (start, end points of holes, and which stack the hole corresponds to). In the worst case, this will be $(2K + 2)$ possible states in a list, as we are keeping the states at the start and end points of all the hole segments and a stack per hole. So, there are $\leq |\mathcal{S}|^{2K+3} \times n^{K+1}$ lists. In the worst case, when there is no $K$-hole bounded run, we may end up generating all possible lists for a given bound $K$ on the hole segments. The time complexity is thus bounded above by $\mathcal{O}(|\mathcal{S}|^{2K+3} \times n^{K+1} + |\mathcal{S}|^5 + |\mathcal{S}|^3 \times |\Delta|)$.

**Beyond Reachability**. We can solve the usual safety questions in the (bounded-hole) underapproximate setting, by checking for underapproximate reachability on the product of the given system with the complement of the safe set. Given the way Algorithm 1 is designed, the fix-point algorithm allows us to go beyond reachability. In particular, we can solve several (increasingly difficult) variants of the repeated reachability problem, without much modification.

Consider the question : For a given state $s$ and MPDA, does there exist a run $\rho$ starting from $s_0$ which visits $s$ infinitely often? This is decidable if we can decompose $\rho$ into a finite prefix $\rho_1$ and an infinite suffix $\rho_2$ s.t. (1) both $\rho_1, \rho_2$ are well-nested, or (2) $\rho_1$ is $K$-hole bounded complete (all stacks empty), and $\rho_2$ is well-nested, or (3) $\rho_1$ is $K$-hole bounded, and $\rho_2 = (\rho_3)^\omega$, where $\rho_3$ is $K$-hole bounded. It is easy to see that (1) is solved by two calls to WellNestedReach and choosing non-empty runs. (2) is solved by a call to Algorithm 1, modified so that we reach $s$, and then calling WellNestedReach. Lastly, to solve (3), first modify Algorithm 1 to check reachability to $s$ with possibly non-empty stacks. Then run the modified algorithm twice : first start from $s_0$ and reach $s$; second start from $s$ and reach $s$ again.

## 4 Generating a Witness

We next focus on the question of generating a witness for an accepting run when our algorithm guarantees non-emptiness. This question is important to address from the point of view of applicability: if our goal is to see if bad states are reachable, i.e., non-emptiness corresponds to presence of a bug, the witness run gives the trace of how the bug came about and hence points to what can be done to fix it (e.g., designing a controller). We remark that this question is difficult in general. While there are naive algorithms which can explore for the witness (thus also solving reachability), these do not use fix-point techniques and hence are not efficient. On the other hand, since we use fix-point computations to speed up our reachability algorithm, finding a witness, i.e., an explicit run witnessing reachability, becomes non-trivial. Generation of a witness in the case of well-nested runs is simpler than the case when the run has holes, and requires us to "unroll" pairs $(s_0, s_f) \in \texttt{WR}$ recursively and generate the sequence of transitions responsible for $(s_0, s_f)$.

**Getting Witnesses from Holes**. Now we move on to the more complicated case of behaviours having holes. Recall that in BFS exploration we start from the states reachable from $s_0$ by well-nested sequences, and explore subsequent states obtained either from (i) a hole creation, or (ii) a pop operation on a stack. Proceeding in this manner, if we reach a final configuration (say $s_f$), with all holes closed (which implies empty stacks), then we declare non-emptiness. To generate a witness, we start from the final state $s_f$ reachable in the run (a leaf node in the BFS exploration tree) and *backtrack* on the BFS exploration tree till we reach the initial state $s_0$. This results in generating a witness run in the reverse, from the right to the left.

• Assume that the current node of the BFS tree was obtained using a pop operation. There are two possibilities to consider here (see below) depending on whether this pop operation closed or shrunk some hole. Recall that each hole has a left end point and a right end point and is of a specific stack $i$, depending on the pending pushes $\downarrow_i$ it has. So, if the MPDA has $k$ stacks, then a list in the exploration tree can have $k$ kinds of holes. The witness algorithm uses $k$ stacks called *witness stacks* to correctly implement the backtracking procedure, to deal with $k$ kinds of holes. Witness stacks should not be confused with the stacks of the MPDA.

• Assume that the current pop operation is closing a hole of kind $i$ as in Figure 2. This hole consists of the atomic holes , and . The atomic hole consists of the push and the well-nested sequence (same for the other two atomic holes). Searching among possible push transitions, we identify the matching push associated with the current pop, resulting in closing the hole. On backtracking, this leads to a parent node with the atomic hole having as left end point, the push , and the right end point as the target of the $ws$ . We push onto the witness stack $i$, a barrier (a delimiter symbol #) followed by the matching push transition and then the $ws$, . The barrier segregates the contents of the witness stack when we have two pop transitions of the same stack in the reverse run, closing/shrinking two different holes.

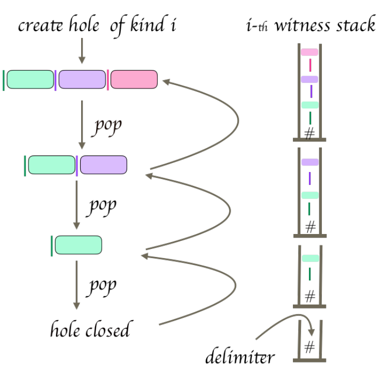

**Figure 2.** Backtracking to spit out the hole in reverse. The transitions of the atomic hole are first written in the reverse order, followed by those of in reverse, and then of in reverse.

• Assume that the current pop operation is shrinking a hole of kind $i$. The list at the present node has this hole, and its parent will have a larger hole (see Figure 2, where the parent node of has ). As in the case above, we first identify the matching push transition, and check if it agrees with the push in the last atomic hole segment in the parent. If so, we populate the witness stack $i$ with the rightmost atomic hole segment of the parent node (see Figure 2, is populated in the stack). Each time we find a pop on backtracking the exploration tree, we find the rightmost atomic hole segment of the parent node, and keep pushing it on the stack, until we reach the node which is obtained as a result of a hole creation. Now we have completely recovered the entire hole information by backtracking, and fill the witness stack with the reversed atomic hole segments which constituted this hole. Notice that when we finish processing a hole of kind $i$, then the witness stack $i$ has the hole reversed inside it, followed by a barrier. The next hole of the same kind $i$ will be treated in the same manner.

• If the current node of the BFS tree is obtained by creating a hole of kind $i$ in the fix-point algorithm, then we pop the contents of witness stack $i$ till we reach a barrier. This spits out the atomic hole segments of the hole from the right to the left, giving us a sequence of push transitions, and the respective $ws$ in between. The transitions constituting the $ws$ are retrieved and added. Notice that popping the witness stack $i$ till a barrier spits out the sequence of transitions in the correct reverse order while backtracking.

## 5 Adding Time to Multi-pushdown systems

In this section, we briefly describe how the algorithms described in section 3 can be extended to work in the timed setting. Due to lack of space, we focus on some of the significant challenges and advances, leaving the formal details and algorithms to the supplement [4]. A TMPDA extends a MPDA $\mathcal{S}$ with a set $\mathcal{X}$ of clock variables. Transitions check constraints which are conjunctions/disjunctions of constraints (called closed guards in the literature) of the form $x \leq c$ or $x \geq c$ for $c \in \mathbb{N}$ and $x$ any clock from $\mathcal{X}$. Symbols pushed on stacks "age" with time elapse; that os, they store the time elapsed since they were pushed onto the stack. A pop is successful only when the age of the symbol lies within a certain interval. The acceptance condition is as in the case of MPDA.

The first main challenge in adapting the algorithms in section 3 to the timed setting was to take care of all possible time elapses along with the operations defined in Algorithm 1. The usage of closed guards in TMPDA means that it suffices to explore all runs with integral time elapses (for a proof see e.g., Lemma 4.1 in [5]). Thus configurations are pairs of states with valuations that are vectors of non-negative integers, each of which is bounded by the maximal constant in the system. Now, to check reachability we need to extend all the precomputations (transitive closure, well-nested reachability, as well as atomic and non-atomic hole segments) with the time elapse information. To do this, we use a weighted version of the Floyd-Warshall algorithm by storing time elapses during precomputations. This allows us to use this precomputed *timed* well-nested reachability information while performing the BFS tree exploration, thus ensuring that any explored state is indeed reachable by a timed run. In doing so, the most challenging part is extending the BFS tree wrt a pop. Here, we not only have to find a split of a hole into an atomic hole-segment and a hole-segment as in Algorithm 1, but also need to keep track of possible partitions of time, making the algorithm quite challenging.

**Timed Witness:** As in the untimed case, we generate a witness certifying nonemptiness of TMPDA. But, producing a witness for the fix-point computation as discussed earlier requires unrolling. The fix-point computation generates a pre-computed set `WRT` of tuples $((s,\nu),t,(s',\nu'))$, where $s, s'$ are states $t$ is time elapsed in the well-nested sequence and $\nu, \nu' \in \mathbb{N}^{|\mathcal{X}|}$ are integral valuations, i.e., integer values taken by clocks. This set of tuples does not have information about the intermediate transitions and time-elapses. To handle this, using the pre-computed information, we define a lexicographic progress measure which ensures termination of this search. The main idea is as follows: the first progress measure is to check if there a time-elapse $t$ transition possible between $(s,\nu)$ and $(s',\nu')$ and if so, we print this out. If not, $\nu' \neq \nu + t$, and some set of clocks have been reset in the transition(s) from $(s,\nu)$ to $(s',\nu')$. The second progress measure looks at the sequence of transitions from $(s,\nu)$ to $(s',\nu')$, consisting of reset transitions (at most the number of clocks) that result in $\nu'$ from $\nu$. If neither the first nor the second progress measure apply, then $\nu = \nu'$, and we are left to explore the last progress measure, by exploring at most $|\mathcal{S}|$ number of transitions from $(s,\nu)$ to $(s',\nu')$. Using this progress measure, we can seamlessly extend the witness generation to the timed setting. The challenges involved therein, can be seen in the full version [4].

## 6 Implementation and Experiments

We implemented a tool BHIM (**B**ounded **H**oles **I**n **M**PDA) in C++ based on Algorithm 1, which takes an MPDA and a constant $K$ as input and returns *True* iff there exists a $K$-hole bounded run from the start state to an accepting state of the MPDA. In case there is such an accepting run, BHIM generates one such, with minimal number of holes. For a given hole bound $K$, BHIM first tries to produce a witness with 0 holes, and iteratively tries to obtain a witness by increasing the bound on holes till $K$. In most cases, BHIM found the witness before reaching the bound $K$. Whenever BHIM's witness had $K$ holes, it is guaranteed that there are no witnesses with a smaller number of holes.

To evaluate the performance of BHIM, we looked at some available benchmarks and modeled them as MPDA. We also added timing constraints to some examples such that they can be modeled as TMPDA. Our tests were run on a GNU/Linux system with Intel® Core™ i7–4770K CPU @ 3.50GHz, and 16GB of RAM. Details of all examples here, as well as an additional example of a linux kernel bug can be found [4].

• **Bluetooth Driver [25]**. The Bluetooth device driver example [25], has an arbitrary number of threads, working with a shared memory. We model this using a 2-stack pushdown system, where a system state represents the current valuation of the global variables, and the stacks are used to maintain the call-return between different functions, as well as to keep track of context switches between threads. A known error as pointed out in [25] is a race condition between two threads where one thread tries to write to a global variable and the other thread tries to read from it. BHIM found this error, with a well-nested witness. A timed extension of this example was also considered, where, a witness was obtained again with hole bound 0.

• **Bluetooth Driver v2** [11,23]. A modified version of Bluetooth driver is considered [11,23], where a counter is maintained to count the number of threads actively using the driver. We model this with a A two stack MPDA. With a well-nested witness, BHIM found the error of interrupted I/O, where the stopping thread kills the driver while the other thread is busy with I/O.

• **A Multi-threaded Producer Consumer Problem**. The producer consumer problem (see e.g., [26]) is a classic example of concurrency and synchronization. An interesting scenario is when there are multiple producers and consumers. Assume that two ingredients called 'A' and 'B' are produced in a production line in batches (of $M$ and $N$ respectively). These parameters $M$ and $N$ are fixed for each day but may vary across days. There is another consumer machine that (1) consumes one unit of 'A' and one unit of 'B' in that order; (2) repeats this process until all ingredients are consumed. In between if one of the ingredients runs out, then we non-deterministically produce more batches of the ingredient and then continue. To avoid wastage the factory aims to consume all ingredients produced in a day, hence the problem of interest is to check if all A's and B's produced in a day are consumed. We can model this factory using a two-stack pushdown system, one stack per product, $A, B$, where the sizes of the batches, $M > 0$ and $N > 0$ respectively, are parameters. The production

| Name | Locations | Transitions | Stacks | Holes | Time Empty (mili sec) | Time Witness (mili sec) | Memory(KB) |
|---|---|---|---|---|---|---|---|
| Bluetooth | 45 | 89 | 2 | 0 | 149.3 | 0.241 | 6934 |
| Bluetooth v2 | 47 | 134 | 2 | 0 | 92.2 | 0.176 | 5632 |
| MultiProdCons(3,2) | 7 | 11 | 2 | 2 | 126.529 | 0.281 | 5632 |
| MultiProdCons(24,7) | 32 | 34 | 2 | 2 | 1879.33 | 10.63 | 21836 |
| Binary Search Tree | 29 | 78 | 2 | 2 | 60.8 | 5.1 | 5143 |
| untimed-$L^{crit}$ | 6 | 10 | 2 | 2 | 14.9 | 0.7 | 4692 |
| untimed-Maze | 9 | 12 | 2 | 0 | 8.25 | 0.07 | 5558 |
| $L^{bh}$ (from Sec. 2.1) | 7 | 13 | 2 | 2 | 22.2 | 0.6 | 4404 |

**Table 1.** Experimental results: Time Empty and Time Witness column represents no. of milliseconds needed for emptiness checking and to generate witness respectively.

and consumption of the 'A's and 'B's are modeled using push and pop in the respective stack. For a given $M$ and $N$, the language accepted by the system is non-empty iff there is a run where all the produced 'A's and 'B's are consumed. The language accepted by the two-stack pushdown system is given by $L_{M,N} = ((a^M + b^N)^+(\bar{a}\bar{b})^+)^+$, where $a, b$ represent respectively, the push on stack 1, 2 and $\bar{a}, \bar{b}$ represent the pop on stack 1, 2 and hence must happen equal number of times.

For any $M, N > 0$, any accepting run of the two stack pushdown system cannot be well-nested. Further, in an accepting run, the minimum number of items produced (and hence its length) must be a multiple of $LCM(M, N)$. As the consumption of 'A's and 'B's happen in an order one by one i.e., in a sequence where consumption of 'A' and 'B' alternate, the minimum number of context changes (and the scope bound) required in an accepting run depends on $M$ and $N$ (in fact it is $O(2 \times LCM(M, N))$. On the other hand, the shortest accepting run is 2-hole bounded: at any position of the word, the open holes come from the unmatched sequences of $a$ and $b$ seen so far. Thus for any $M, N{>}0$, BHIM was able to check for non-emptiness of $L_{M,N}$ with a witness of hole bound 2.

• **Critical time constraints** [9]. This is one of the timed examples, where we consider the language $L^{crit} = \{a^y b^z c^y d^z \mid y, z \geq 1\}$ with time constraints between occurrences of symbols. The first $c$ must appear after 1 time-unit of the last $a$, the first $d$ must appear within 3 time-units of the last $b$, and the last $b$ must appear within 2 time units from the start, and the last $d$ must appear at 4 time units. $L^{crit}$ is accepted by a TMPDA with two timed stacks. $L^{crit}$ has no well-nested word, is 4-context bounded, but only 2 hole-bounded.

• **Concurrent Insertions in Binary Search Trees**. Concurrent insertions in binary search trees is a very important problem in database management systems. [17,11] proposes an algorithm to solve this problem for concurrent implementations. However, incorrect implementation of locks allows a thread to overwrite others. We modified the algorithm [17] to capture this bug, and modeled it as MPDA. BHIM found the bug with a witness of hole-bound 2.

• **Maze Example**. Finally we consider a robot navigating a maze, picking items; an extended (from single to multiple stack) version of the example from [6]. In the untimed setting, a witness for non-emptiness was obtained with hole-bound 0, while in the extension with time, the witness had a hole-bound 2.

| Name | Locations | Transitions | Stacks | Clocks | $c_{max}$ | Aged(Y/N) | Holes | Time Empty(mili sec) | Time Witness (mili sec) | Memory(KB) |
|---|---|---|---|---|---|---|---|---|---|---|
| Bluetooth | 45 | 89 | 2 | 0 | 2 | Y | 0 | 152.8 | 0.119 | 5568 |
| $L^{crit}$ | 6 | 10 | 2 | 2 | 8 | Y | 2 | 9965.2 | 3.7 | 203396 |
| Maze | 9 | 12 | 2 | 2 | 5 | Y | 2 | 349.3 | 0.31 | 11604 |

**Table 2.** Experimental results of timed examples. The column $c_{max}$ is defined as the maximum constant in the automaton, and Aged denotes if the stack is timed or not

**Results and Discussion**. The performance of BHIM is presented in Table 1 for untimed examples and in Table 2 for timed examples.

Apart from the results in the tables, to check the robustness of BHIM wrt parameters like the number of locations, transitions, stacks, holes and clocks (for TMPDA), we looked at examples with an empty language, by making accepting states non-accepting in the examples considered so far. This forces BHIM to explore all possible paths in the BFS tree, generating the lists at all nodes. The scalability of BHIM wrt all these parameters are in [4].

BHIM **Vs. State of the art**. What makes BHIM stand apart wrt the existing state of the art tools is that (i) none of the existing tools handle underapproximations captured by bounded holes, (ii) none of the existing tools work with multiple stacks in the timed setting (even closed guards!). The state of the art research in underapproximations wrt untimed multistack pushdown systems has produced some robust tools like GetaFix which handles multi-threaded programs with bounded context switches. While we have adapted some of the examples from GetaFix, the latest available version of GetaFix has some issues in handling those examples[3]. Likewise, SPADE, MAGIC and the counter implementation [16] are currently not maintained, resulting in a non-comparison of BHIM and these tools. Most examples handled by BHIM correspond to non-context bounded, or non-scope bounded, or timed languages which are beyond GetaFix : the 2-hole bounded witness found by BHIM for the language $L_{9,5}$ for the multi producer consumer case cannot be found by GetaFix/MAGIC/SPADE with less than 90 context switches. In the timed setting, the Maze example which has a 2 hole-bounded witness where the robot visits certain locations equal number of times is beyond [6], which can handle only single stack.

## 7 Future Work

As immediate future work, we are working on BHIM **v2** to be symbolic, inspired from GetaFix. The current avatar of BHIM showcases the efficiency of fix-point techniques extended to larger bounded underapproximations; indeed going symbolic will make BHIM much more robust and scalable. This version will also include a parser to handle boolean programs, allowing us to evaluate larger repositories of available benchmarks.

*Acknowledgements.* We would like to thank Gennaro Parlato for the discussions on GetaFix and for providing us benchmarks and anonymous reviewers for more pointers.

[3] we did get in touch with one of the authors, who confirmed this.
